# University of Buffalo

**Published:** 1857-10

**Authors:** 


*University of Buffalo.*—The announcement of this school in the present number, reminds us of our delinquency in not referring renewedly to its claims upon our profession and the attraction it holds out to students. Its faculty, including such names as Hamilton, Flint, White, Hunt, Lee, and Rochester, whom we know, and their colleagues, all of whom are able and practical teachers, places this medical college on a par with any one in our state or country. The college and hospital buildings offer clinical opportunities for students, unsurpassed, whole the location is at a point accessible from a wide extent of our own country and adjacent to the Canadas. We shall be surprised if this school does not hereafter exceed any former prosperity.—*Am. Med. Gaz.*

Dr. Reese, editor of the American Medical Gazette, has paid, in the above, a glowing compliment, indeed, to one of our city institutions. But is the compliment deserved? The University is embarrassed with its own dreadful unpopularity with the medical public. For years past it has hardly made a show of students. We believe, too, that the disfavor which it suffers is mainly owing to *some* or most of its managers. We can assure the editor of the Gazette, if this school does hereafter exceed any former prosperity, it will be only under an entirely new administration, and not from any recuperative power which it now possesses.—*Buffalo* "*Advocate.*"

We regret to see in a paper, claiming to possess more than the usual modicum of Christian spirit so malignant an attack on an institution which is, and should be, esteemed and prized by all who have any means of judging of its character and usefulness. Local pride alone should have prevented such a tirade; an ordinary sense of justice to men who have labored long and with small reward, in the support of an institution of learning, should have forbidden the publication of a paragraph highly injurious to their personal reputations.

The Advocate says that the "University is embarrassed with its own dreadful unpopularity with the medical public." But the Advocate cannot name ten medical men who will speak in anything but terms of praise of this school; and although the University has been secondarily involved in exciting controversy with some of the medical journals of the country, not one of them has ever dared to utter one word against the character of its faculty, as men or teachers.

The Advocate says that "for years past it has made scarcely a show of students." Let us see how this is. Last winter it had between fifty and sixty *bona fide* students. The neighboring school at Cleveland, which once had 250, had then only thirty students. At Geneva, where the class some years since, *and under the conduct of some of the same men now engaged in the Buffalo school*, numbered 200, they had last winter but seven. At Columbus, Ohio, with a building costing $70,000, and every other attraction, the class numbered about forty. In Cincinnati no one of the three paid expenses.

The reason for this state of things is two-fold. The profession of medicine *pays* so poorly that fewer young men than formerly choose it as a means of livelihood; and, second, the schools of New York and Philadelphia have made every effort to increase the well-known tendency to centralization in those cities; so that now comparatively few young men graduate in provincial schools. Under these two discouraging causes, the Buffalo Medical College has maintained itself better than any other of the so-called country schools, because it has a better museum, better hospital advantages, and better teachers than they.

As to the prospect of any "new administration" in the Buffalo school, we can assure our friend of the Advocate, that it may abandon all hope. Its faculty and council are a unit in all their purposes. Its teachers are, with perhaps one exception,—personal modesty forbids the mention of a name,—men of reputation. For a series of years the medical works they have published have been standards, and their contributions to the Transactions of the American Medical Association have been the leading features of its meetings. The names of at least three of them are well known and frequently quoted in the highest medical circles of France and England; the books of one of them have been reviewed in tones of eulogy by the highest authority in both those countries; and the faculty feel that so long as they possess the confidence and esteem of the profession of the country at large, as well as that of Europe, they can stand up under the criticism of the Advocate.—*Buffalo Commercial Advertiser.*

---

*Fees for consultations by letter.*—MESSRS. EDITORS: Will you give insertion to the following extract from the Boston Medical & Surgical Journal, for the benefit of the members of the profession in this region who belong to the band of "afflicted brothers" to which Xenophon alludes? All sensible readers must concur in the reasonableness of the views expressed by the Boston editors:

*Medical Ethics.—A letter of advice equivalent to a consultation, and should be in like manner remunerated.*—MESSRS. EDITORS: Suppose you were to receive a letter, filling full three pages of fair foolscap, and reciting all the facts (to the writer) of a case, reporting the treatment, and finally asking an opinion and an advice—but containing neither fee nor postage stamp. What would you do? Would you read it? Suppose you do read it, but can really make no opinion, either of diagnosis, prognosis or treatment. *Would you answer it?*

Again: Suppose you receive another letter from another source, *without any enclosure.* Suppose you *can* form an opinion about the disease, and might recommend a treatment. Do you feel bound to answer it?

One largely afflicted brother in this way, impatiently waits an answer.

XENOPHON.

With regard to Xenophon's first question, we think the practitioner is not bound to take notice of a letter containing neither fee, postage stamp, nor an intelligible account of the case. He may, if he pleases, put the letter in the fire. We should read it, but should not, under ordinary circumstances, answer it.

As to the second query, we should withhold an opinion until the fee were paid, if the request came from an unknown party. We should not feel bound to answer it.

A person writing a description of a case, and requesting in answer an opinion or advice, is bound to enclose in his letter the usual fee, or to ask the amount owing, and to transmit it by return mail. (The fee for a letter of advice, established by the Boston Medical Association, is from *five* to *ten dollars.*) It is always understood that a business letter requiring an answer (except between regular correspondents,) should contain a postage stamp.

We state our opinion in general; of course there are exceptions. A former pupil or a personal friend has a right to ask an opiniou without being expected to pay for it; but this privilege is not to be abused, especially in a case really requiring a consultation, the patient being able to pay. When the patient is too poor to pay the fee, if this is distinctly stated, the party giving the opinion will ask no compensation. In short, a letter of advice is the same thing as a consultation, and the writer is not only entitled to his fee, but ought to insist on receiving it, where the advice is regularly sought, and the patient able to pay.—*Boston Med. & Surg. Jour.*

---

CLEVELAND, Sept. 8th, 1857.

Editor Buffalo Medical Journal.

SIR: I send you a *special notice*, taken from the circular of the Cleveland Med. College, calling the attention of the medical profession, to the fact that Prof. Weber is the medical superintendent of the Cleveland Infirmary, (which means the Cleveland Poorhouse) claiming valuable clinical advantages afforded by that institution, to such students as attend the Cleveland College. Now the said Infirmary or Poorhouse, is over three (3) miles from the college; I would like to know how the students are to be particularly benefitted; are the sick to be brought a distance of over three miles, in the winter months, or are the students to visit the poorhouse, for clinical instruction? I presume the latter, and I would suggest to the faculty, that they charter *one* of Mr. Stevens' omnibusses, for the purpose of carrying the medical class and themselves to said institution on clinic days.

By looking over this special notice, you will see that the faculty also announce that through the politeness of Dr. Tod, the class will be permitted to visit the U. S. Marine Hospital for the purpose of clinical instruction. If Dr. Tod has given such a pledge, he has overstepped his privileges, and I will venture the assertion that the Secretary of the Treasury will not permit it.

I wish to inform the medical profession how Prof. Weber obtained the appointment of physician to the Cleveland Poorhouse, and I wish it be understood that the faculty of the Cleveland College fully endorse his conduct in this matter.

The city paid last year to the physician eight hundred dollars as a salary, and all agree that the amount was sufficiently low; this year there was a number of applicants for the post, as is usual, among whom there were several graduates of the Cleveland Medical College; none were willing to do the business for a less sum than eight hundred dollars, except our recently imported German Professor, who meant to make a sure thing of it, and put in his bid at the *modest* sum of two hundred dollars, cutting down the fees six hundred dollars at one dash. Surely their own graduates and the profession generally, are under vast obligations to this school. It remains to be seen whether the medical profession will sustain a school whose faculty condescend to such unmitigated meanness, and unprofessional conduct. If this kind of conduct meets with the approval of our medical colleges, the sooner they are wound up the better it will be for the profession. I have no hesitation in saying that all such miserable catch-penny schools tend only to degrade the profession instead of elevating it. If their conduct does not forfeit all claims to respectability I do not know what can.

A PHYSICIAN OF CLEVELAND.

*Special Notice.*—Professor Weber, being now Medical Superintendent of the Cleveland City Infirmary, the valuable clinical advantages afforded by that institution, which always contains a large number of patients, will be fully open to the medical class.

Also, by the politeness of Dr. Tod, the present medical officer of the U. S. Hospital, located in this city, we are further permitted to assure students who may find it convenient to attend the lectures in Cleveland Medical College, that arrangements for extending their privileges in clinical instruction, will also be made at the hospital; subject, of course, to such regulation as the U. S. Government may prescribe.

In the September number of the Southern Medical and Surgical Journal, we find an account of a novel method of treating intestinal obstruction, with the report of a case by Dr. Tate, of West Point, Ga. Our space will not permit us to give the entire article, but we can merely describe his new method with the result, in the reported case, which was entire relief after all other measures had failed.

Dr. Tate was called to see a negro man who had been suffering for some time from constipation. All sorts of means were employed to procure an evacuation, but without effect; the next day, however, he discovered a strangulated inguinal hernia, which was successfully operated on on the following morning. To the doctor's surprise, the bowels did not move after the operation, in spite of the administration of croton oil, castor oil, and the injection of a gallon of water. The doctor then decided on the following course of treatment, which we insert:

March 18th, 2 o'clock, A. M. Being well satisfied that an intussusception or rather mechanical obstruction, existed above the strangulated point, and having, as I conceived, used every remedy worthy of trial in such a case, I determined to proceed upon my own responsibility, let consequences be as they might; therefore, I began again the use of warm water enemas, throwing them into the bowels slowly and cautiously; and after having introduced by a pump syringe, one gallon of water, I next dissolved 40 grs. of *tartaric acid* in ℥iv of water, introduced that into the intestines; had a large compress prepared and placed in the hands of a strong negro fellow, with instructions to apply it to the *anus*, and hold it there, so as effectually to prevent the escape of either gas or water after I should introduce 40 grs. of *bi-carb. of soda*, dissolved also in ℥iv of water. The soda was introduced, the compress used admirably, and poor Will rolling on the floor, crying at the top of his voice, "I shall burst—take that thing away, my bowels are tearing in two." The compress was removed; gas, water and fæcal matter escape freely, to the astonishment of all bystanders. In half an hour the same amount of warm water, tartaric acid and soda were used again, and with the same happy effect.

The only medicine given after this, was calcined charcoal, which passed through his bowels with no difficulty. All being well satisfied that the obstruction was fully overcome, and Will declaring himself cured, he was discharged on the 20th.

The introduction of large quantities of water through a long tube is not novel, and occasionally we introduce air in the same way; but the proposition of Dr. Tate to use gas generated in the intestines, is new and worthy of trial. It reminds us of the story of an Eastern Cadi, who captured a stranger and some seidletz powders, which he was assured possessed remarkable virtue. Accordingly he swallowed the contents of all the white papers, following them immediately by all the blue; what the result was is not recorded, but our readers can imagine what a *reäction* must have followed. F.

